# Root canal morphology of the mandibular second premolar: a systematic review and meta-analysis

**DOI:** 10.1186/s12903-021-01668-z

**Published:** 2021-06-16

**Authors:** Thomas Gerhard Wolf, A. L. Anderegg, R. J. Wierichs, G. Campus

**Affiliations:** 1 Department of Restorative, Preventive and Pediatric Dentistry, School of Dental Medicine, University of Bern, Freiburgstrasse 7, 3010 Bern, Switzerland; 2 Department of Periodontology and Operative Dentistry, University Medical Center of the Johannes Gutenberg-University Mainz, Mainz, Germany; 3 Department of Surgery, Microsurgery and Medicine Sciences, School of Dentistry, University of Sassari, Sassari, Italy; 4 School of Dentistry, Sechenov University, Moscow, Russia

**Keywords:** Internal morphology, Mandibular second premolar, Number of roots, Number of root canals, Root canal configuration, Systematic review

## Abstract

**Background:**

The aim of this paper was to systematically review the root canal configuration (RCC) and morphology literature of the mandibular second premolar (Mn2P).

**Methods:**

Systematic research of five electronic databases was performed to identify published literature concerning the root canal configuration (RCC) of the Mn2P up through July 2020. Studies were selected according to predefined search terms and keywords inclusion criteria: “root canal configuration”, “root canal system”, “root canal morphology”, “mandibular second premolar”, “mandibular premolars”, “morphology” and “anatomy”. Further possible studies were identified by cross-referencing and screening the bibliographies of the selected articles.

**Results:**

From 1622 retrieved studies, 44 studies investigating the internal morphology of 17,839 Mn2Ps were included. Most examined Mn2Ps were single-rooted (89.5–100%); two-rooted (0.1–8%) and three-rooted (0.1–3.5%) Mn2Ps at lower frequency. Most frequent RCCs reported were 1–1–1/1 (55.3–99.6%) followed by 1–1–2/2 (0.5–57%) and 2–2–2/2 (0.6–18%). The meta-analysis of seven studies demonstrated that a significantly higher number of RCC type 1–2–1/1 (OR [95%CI] = 2.05 [1.27, 3.33]) and 2–2–2/2 (OR [95%CI] = 2.32 [0.65, 8.63]) were observed in male than in female patients.

**Conclusions:**

Different RCC research methods have been reported. Whereas clearing and radiographs were commonly used in the past, CBCT has been prevalent in recent years. A globally high frequency of a 1–1–1/1 RCC in the Mn2P has been reported. Nevertheless, the probability that different, more complicated RCCs can appear in Mn2Ps should not be underestimated and, thus, should be taken into consideration when making decisions during an endodontic treatment.

**Supplementary Information:**

The online version contains supplementary material available at 10.1186/s12903-021-01668-z.

## Background

The most significant causes for endodontic failure are incomplete instrumentation followed by incorrect obturation of the root canal space [1]. Lack of root canal morphology knowledge is a consequential hindrance for meticulous cleaning, shaping and obturation of the root canal system of a tooth needing endodontic treatment. In daily endodontic practice, the dental practitioner is confronted with such factors as root canal number, size, and shape, which results in dimension making [2–43]. The root canal system configuration (RCC) of mandibular second premolars (Mn2P) is typically described as a single-rooted tooth with a 1–1–1/1 RCC according to the classification described by Briseño Marroquín et al. [44]. However, a sizeable variation in the number of roots and root canals of the Mn2P was described in which the internal root canal morphology can be quite diversified [6, 29, 45].

A number of RCC investigations of the Mn2P have been carried out and analyzed with different research methodologies such as clearing [4, 8, 14, 17, 28, 32, 36, 39], optical augmentation [32, 44], cross-sectioning [5], radiography [5, 25, 30, 38, 41, 43, 46], CBCT [2, 3, 6, 9–12, 15–24, 26, 27, 29, 33, 34, 37, 42, 47] and dental computed tomography [41]. To the best of authors’ knowledge, the root canal morphology of Mn2Ps by means of micro-computed tomography (micro-CT) has not been reported. Micro-CT has been described as a reproducible, non-destructive and non-invasive high-resolution *ex vivo* method that, in association with 3D software imaging, is actually considered the most accurate root canal morphology research method [48] as well as the gold standard in endodontic internal morphology research [49]. The most frequently used root canal classification systems of Vertucci [40] and Weine et al. [50] are frequently reported; however, they are limited when describing an individual root canal morphology with precision, especially in cases of a complex root canal system. Therefore, a four-digit RCC system was created by Briseño Marroquín et al. [44]; the advantage of this RCC-system classification is that the classification system is a descriptive one and can be individually applied to the internal morphology of a specific tooth rather than forcing a classification based on the internal morphology system. The aim of this investigation was to undertake a systematic review of the literature concerning the root canal configuration of mandibular second premolars.

## Methods

**Fig. 1:**
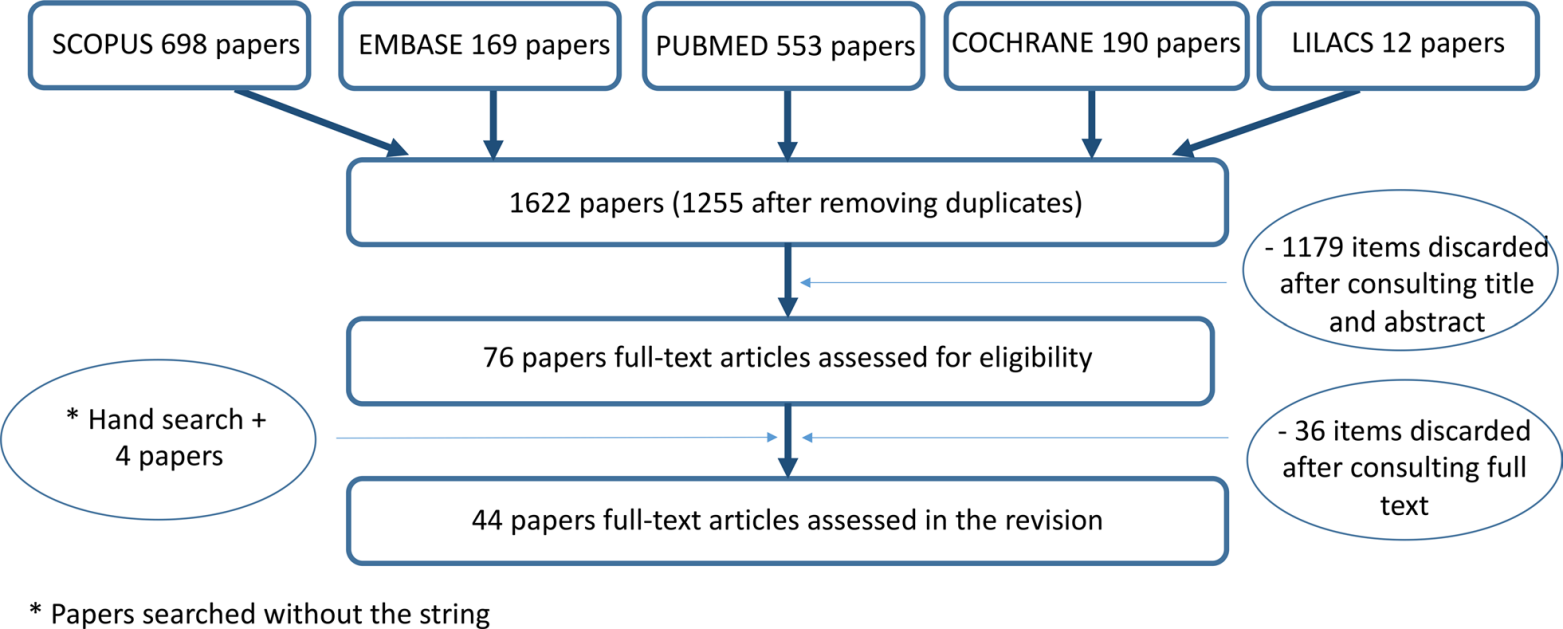
Flowchart of the literature search and selection process. The references were retrieved from the databases Cochrane Database, Embase, Lilacs, MEDLINE/PubMed and Scopus (*studies searched without a string)

A systematic review to identify published literature concerning the root canal configuration (RCC) of the mandibular second premolar (Mn2P) until the end of July 2020 was carried out through a reference search of five electronic databases (Cochrane Database, Embase, MEDLINE/PubMed, Lilacs and Scopus) (Fig. 1). The current systematic review follows the Preferred Reporting Items for Systematic Reviews and Meta-Analyses (PRISMA) guidelines [51]. The review protocol was registered in the international prospective register of systematic reviews (PROSPERO) system (CRD42020192030, 14 July 2020).

Randomized controlled trials, cross-sectional, comparative, validation and evaluation studies of RCC’s of Mn2Ps of different populations in patients of any age were included. Using a standardized comprehensive search strategy, the following Medical Subject Heading (MeSH) terms and keywords were used: “Root canal configuration” OR “root canal system” OR “root canal morphology” AND “mandibular second premolar” OR “mandibular premolars” AND “morphology” OR “anatomy”. Additionally, other related studies were added by cross-referencing and hand searching the bibliographies of full text articles. The data collection was performed by an ad hoc-designed data extraction form without masking bibliographic record data, title, or authors. Only articles in English were considered. Studies in which teeth were only described as premolars or mandibular premolars without a clear assignment as well as case reports were excluded. After comparing the results from the five databases and the hand search, duplicates or repeated articles were rejected. Title and abstracts of the received articles were examined by two independent reviewers (A.L.A., T.G.W.) and if deemed relevant, the corresponding full text articles were consulted. Publication year and study duration, details/characteristics of the participants at baseline, and data regarding the RCC were recorded when available. The corresponding results, including relevant aspects, were summarized in tables. The obtained articles’ abstracts, establishing whether the article should be excluded or included in the systematic review, were examined by two independent reviewers (A.L.A., T.G.W.). Thus, articles not matching the inclusion criteria were excluded. All articles meeting the inclusion criteria were retrieved in pdf format. The frequency of root canal configurations, the number of teeth, the number of roots, and the place of origin of the samples studied were presented in tables using the classifications of Vertucci [40], Weine et al. [50], and Briseño Marroquín et al. [44]. Briseño Marroquín et al. [44] RCC describe the root internal morphology in a coronal, middle and apical third direction by means of a four digits system. The first three digits are separated with a dash and represent the root canal number at the coronal boundary of the coronal, middle and apical third, respectively. The fourth digit is separated from the other three numbers with a slash and represents the number of apical main portals of exit. In addition, the different laboratory research methodologies that have been used by the different investigation groups were summarized in the tables as well.

The quality assessment of the included RCCs were assessed by two independent reviewers (A.L.A., T.G.W.) following the customized quality assessment tool developed by the National Heart, Lung, and Blood Institute (www.nhlbi.nih.gov/health-topics/study-quality-assessment-tools). In case of disagreements between the independent reviewers, this has been discussed. If no consensus could be achieved, a third reviewer (R.J.W.) was consulted.

The risk of bias was assessed using the anatomical quality assessment (AQUA) tool for the quality assessment of anatomical studies included in meta-analyses and systematic reviews [52]. The same two authors (A.L.A., T.G.W.) screened the articles assessing the risk of bias; in case of disagreement in the assessment, the same author (R.J.W.) was consulted to reach consensus.

The Review Manager software (RevMan version 5.4 software, Cochrane Collaboration, Copenhagen, Denmark, 2014) was applied for the statistical analyses of the papers included into the meta-analyses. Odds ratio (OR) were chosen for calculating the effect size. The I^2^ statistic was calculated to describe the percentage of variation across studies due to heterogeneity rather than chance [53]. Fixed or random-effects meta-analysis was performed depending on heterogeneity (I^2^ < 35%: fixed-effects; I^2^ > 35%: random-effect) [54, 55]. The primary measures of effect between different root canal configurations, patient’ sex and geographic reasons were Odds ratio and 95% confidence intervals (95% CI) for studies using dichotomous outcome data. Statistical significance was defined as a *p* value ≤  0.05.

## Results

The literature search of five different databases identified 1622 papers. After the results were compared and all duplicates were removed, 1255 articles were left in the initial search. Seventy-six studies that met the inclusion criteria were further considered after the title and abstract were consulted. After a full text analysis and adding articles retrieved by hand search, a total of 44 studies were included in this review (Fig. 1). These 44 morphology studies examined a total of 17,839 mandibular second premolars (Mn2Ps). The investigations included were carried out in different regions of the world and with different research methodologies.

**Fig. 2:**
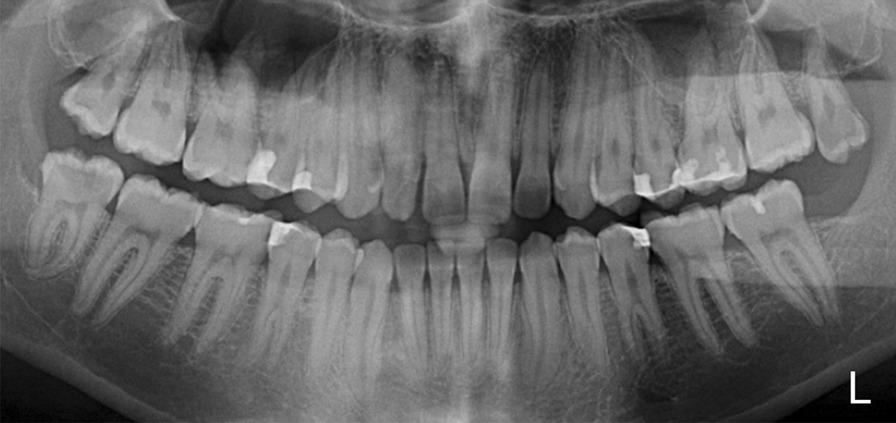
Coincidental observation in a panoramic radiograph section of a male Swiss individual depicting bilateral two-rooted mandibular second premolars. Kannan et al. [56] described a similar clinical case with contralateral two-rooted mandibular second premolars in an Indian individual. Multiple root canals can also be presumed in the second maxillary and first mandibular premolars

The results are divided into authors, year and reference number, population, number of teeth investigated, research method employed, root canal configuration frequency (%) and number of roots (%) (Table 1). It could be observed that most of the investigated Mn2Ps were single-rooted (89.5–100%) [2, 3, 6, 7, 10, 11, 14–16, 18, 20–24, 27–29, 31–35, 37–39, 41, 42, 46, 47], followed by two-rooted Mn2Ps with a frequency lower than 8% [2, 3, 6, 7, 10, 11, 14, 20–23, 29, 33, 34, 38, 39, 41, 46, 47], while three roots were reported only in 0.1 to 3.5% [2, 7, 33]. A 1–1–1/1 RCC is the most frequently observed classification in Mn2Ps (Vertucci’s and Weine’s Type I) with a frequency up to 99.6% [21]. The second most often RCC reported in Mn2Ps (57.1%) is the 1–1–2/2 [7] (Vertucci’s Type V), whereas Weine et al. [50] do not describe this RCC. Briseño Marroquín’s RCC type 2–2–2/2 (Vertucci’s Type IV; Weine’s Type III) has been frequently observed in the reviewed studies. Among the summarized studies in Table 1, other RCCs such as 2–2–1/1 (Vertucci’s and Weine’s Type II), 1–2–1/1 (Vertucci’s Type III), 2–1–2/2 (Vertucci’s Type VI), 1–2–1–/2 (Vertucci’s Type VII) and 1–1–3-/3 (Vertucci’s Type VIII) were observed less frequently. Comparative gender difference studies are summarized in Table 2. A higher [15, 22, 24, 33, 36, 47] or similar [2] frequency of the 1–1–1/1 RCC in female individuals has been reported. A second root in Mn2Ps has been also reported [2, 3, 6, 7, 10, 11, 14, 20–23, 29, 33, 34, 38, 39, 41, 47] with a frequency from 0.1 to 8.0% (Fig. 2).

**Table 1:** Systematic literature review summary of different comparative and non-comparative morphologic investigations of the root canal configuration (RCC) of mandibular second premolars

Report		PP	n	Met	RCC-frequency (%)									Roots (n; %)		
	Q/R	RCC		Ve (1984)	I	II	III	IV	V	VI	VII	VIII	*	1	2	3
				We (1969)	I	II		III					*			
				Br (2015)	1–1–1/1	2–2–1/1	1–2–1/1	2–2–2/2	1–1–2/2	2–1–2/2	1–2–1/2	1–1–3/3	*			
Pineda and Kuttler [30]	G	MEX	250	Rx	98.8	–	–	1.2	–	–	–	–	–	–	–	–
Green et al. [13]	G	USA	50	Gr	92.0	–	–	8.0	–	–	–	–	–	–	–	–
Zillich and Dowson [43]	G	USA	938	Rx	84.5	0.9	0	10.8	0	0	0	0	0	–	–	–
Miyoshi et al. [25]	F	JPN	653	Rx	97.9	–	-	2.1	–	–	–	–	–	–	–	–
Vertucci [40]	G	USA	400	Cl; He	97.5	0	0	0	2.5	0	0	0	0	–	–	–
Sikri and Sikri [38]	G	IND	96	Rx; Cr	86.5	0	0	2.0	11.5	0	0	0	0	97.9	2.1	0
Calişkan et al. [8]	G	TUR	100	Cl	93.6	–	–	–	6.38	–	–	–	–	–	–	–
Sert and Bayirli [36] Comp	G	TUR	100	Cl; In M	57.0	7.0	7.0	18.0	6.0	3.0	2.0	0	0	–	–	–
			100	F	85.0	7.0	0	0	8.0	0	0	0	0	–	–	–
Rahimi et al. [32]	G	IRN	103	Cl; In	76.3	7.9	9.9	5.9	0	0	0	0	0	100	0	0
Awawdeh and Al-Qudah [4]	F	JOR	400	Cl	72.0	3.8	1.0	7.5	15.3	0	0	0	0.5	–	–	–
Rahimi et al. [31]	G	IRN	137	Cl; In; 5x	89.8	1.46	2.92	3.64	0	0	0	0	0.73	100	0	0
Parekh et al. [28]	F	IND	40	Cl	80.0	0	0	2.5	17.5	0	0	0	0	100	0	0
Yu et al. [42]	G	CHN	178	CBCT	97.2	0.55	0	0	1.7	0	0	0	0.55	100	0	0
Bolhari et al. [5] Comp	F	IRN	217	Rx; m-d	92.9	2.76	2.76	0.46	1.38	0	0	0	0	–	–	–
				Rx; b-l	94.4	1.38	2.3	0.46	1.38	0	0	0	0	–	–	–
				Cr; 40x	91.4	3.22	1.84	1.38	1.38	0	0	0	0.9	–	–	–
Salarpour et al. [35]	G	IRN	41	CBCT	75.6	–	–	–	22	–	–	–	2.4	100	0	0
Yadav et al. [41]	G	IND	310	Rx; dental CT	93.2	–	–	3.8	–	–	–	1.9	0.6	93.5	6.1	0.3
LLena et al. [18]	G	ESP	53	CBCT	90.6	1.8	–	–	7.5	–	–	–	–	100	0	0
Ok et al. [26]	G	TUR	1345	CBCT	98.5	0.1	0.07	0.6	0.5	0	0	0.2	–	–	–	–
Shetty et al. [37]	G	IND	814	CBCT	93.5	1.4	0.2	0	3.9	0	0	0.1	0.7	100	0	0
Singh and Pawar [39]	F	IND	100	Cl; In	66	30.0	–	–	4.0	–	–	–	–	92	8.0	0
Arslan et al. [3]	G	TUR	133	CBCT	92.4	2.2	0.7	0	1.5	0	0	0	2.9	96.2	3.8	0
Bulut et al. [6]	F	TUR	549	CBCT	98.9	0.2	0.4	0	0.5	0	0	0	0	98.9	1.1	0
Felsypremila et al. [12]	G	IND	398	CBCT	98.4	0	0	0	0.8	0	0	0	0.8	–	–	–
Habib et al. [14]	P	SYR	65	Cl; In	83.1	10.8	0	4.62	0	0	0	1.53	0	96.9	3.1	0
Çelikten et al. [9]	G	TUR	433	CBCT	96.6	1.1	1.1	–	1.1	–	–	–	–	–	–	–
Bürklein et al. [7]	G	GER	871	CBCT	39.0	1.1	0.1	1.4	57.1	0.5	0.3	0.3	0	98.6	1.3	0.1
Hajihassani et al. [15] Comp	P	IRN	57	CBCT F	80.7	1.8	7.0	0	8.8	1.8	0	0	0	100	0	0
			43	M	74.7	4.7	16.3	0	4.7	0	0	0	0	100	0	0
Khademi et al. [17] Comp	P	IRN	182 (Ma2P & Ma1P)	Cl; In M	87.9	1.1	0	2.2	8.8	0	0	0	0	–	–	–
				CBCT	92.3	0	2.2	2.2	3.3	0	0	0	0	–	–	–
Martins et al. [20]	G	PRT	833	CBCT	95.7	0.8	1.3	0.5	1.4	–	–	–	–	99.9	0.1	0
Martins et al. [23]	G	PRT	821	CBCT	95.8	0.8	1.2	0.5	1.3	0	0	0	0.3	99.9	0.1	0
Martins et al. [21] Comp	G	CHN	235	CBCT	99.6	0.4	0	0	0	0	0	0	0	100	0	0
		PRT	858		95.7	0.8	1.3	0.5	1.4	0	0	0	0.3	99.9	0.1	0
Martins et al. [22] Comp	G	PRT	331	CBCT M	94.3	0.6	2.1	0.6	1.5	0	0	0	0.9	99.7	0.3	0
			527	F	96.6	0.9	0.8	0.4	1.3	0	0	0	0	100	0	0
Martins et al. [19] Comp	G	PRT (age ≤ 20)	13	CBCT	69.2	0	7.7	0	7.7	0	0	0	15.4	–	–	–
			251	21–40	98.8	0	0.4	0	0.8	0	0	0	0	–	–	–
			395	41–60	96.2	1.3	1.3	0.2	0.8	0	0	0	0.2	–	–	–
			199	≥ 61	92.5	1.0	2.0	1.5	3.0	0	0	0	0	–	–	–
Pedemonte et al. [29] Comp	G	BEL	101	CBCT	92.1	–	3.0	–	5	–	–	–	1	98.0	2.0	0
		CHL	100		95.0	–	2.0	–	2	–	–	– –	0	99.0	1.0	0
Razumova et al. [34]	F	RUS	443	CBCT	90.1	.	.	9.9	–	–	–	–	–	99.8	0.2	0
Alfawaz et al. [2]	G	SAU	172	CBCT F	90.1	3.5	0	1.7	1.2	0	0	3.5	0	95.3	3.5	1.2
			171	M	90.1	5.3	0.6	3.5	0.6	0	0	0	0	95.9	4.1	0
Corbella et al. [10]	G	ITA	100	CBCT	95.0	0	1.0	4	0	0	0	0	0	97	3.0	0
Corbella et al. [11]	G	ITA	88	CBCT	95.5	0	0	4.5	0	0	0	0	0	96.6	3.4	0
Kaya Buyukbayram et al. [16]	G	TUR	264	CBCT	97.7	0	1.1	0	0.38	0	0	0	0.8	100	0	0
Mashyakhy and Gambarini [24] Comp	G	SAU	188	CBCT M	94.7	0	2.7	0	1.6	0	0	0	1.1	100	0	0
			191	F	99.0	0	0.5	0	0	0	0	0	0.5	100	0	0
Pan et al. [27]	G	MYS	399	CBCT	99.5	0.3	–	0.3	–	–	–	–	–	100	0	0
Rajakeerthi and Nivedhitha [33] Comp	G	IND	200	CBCT M	55.3	8.8	6.1	4.4	15.8	3.5	1.8	4.4	0	89.5	7.0	3.5
				F	57	7	8.1	5.8	9.3	2.3	5.8	4.7	0	90.7	5.8	3.5
Kharouf et al. [46]	G	FRA	56	Rx, *in vivo*	76.8	–	–	12.5	–	–	–	–	10.7	–	–	–
Shemesh et al. [47]	G	ISR	1678 (M 831/F 84)	CBCT M	96.4	0.6	1.7	0.1	0.6	0	0	0.1	0.5	99.5	0.5	–
				F	97.6	0.1	1.1		0.5	0	0	0.3	0.2	99.8	0.2	–

The RCCs are depicted according to the classifications of Weine et al. (We) [50], Vertucci (Ve) [40] and Briseño Marroquín et al. (Br) [44]

*Comp* comparative study design, *Met* research methodology employed, *Q/R* quality rank, *PP* country 3-digit code of population investigated, * no classification given/possible, *Cl* clearing method, *In* India ink dye, *He* hematoxylin dye, *Rx* radiographic method, *Gr* grinding method, *Mic* stereo microscopic method, *Cr* cross/sectional method, *CBCT* cone-beam computed tomography, *F* female, *M* male, *m-d* mesio-distal, *b-l* bucco-lingual

**Table 2:** Summary of mandibular second premolars studies recording gender differences

Report	PP	n	Met	RCC-frequency (%)									Roots (n; %)		
	RCC		Ve (1984)	I	II	III	IV	V	VI	VII	VIII	*	1	2	3
			We (1969)	I	II		III					*			
			Br (2015)	1–1–1/1	2–2–1/1	1–2–1/1	2–2–2/2	1–1–2/2	2–1–2/2	1–2–1/2	1–1–3/3	*			
Sert and Bayirli [36] Comp	TUR	100	Cl; In M	57.0	7.0	7.0	18.0	6.0	3.0	2.0	0	0	–	–	–
		100	F	85.0	7.0	0	0	8.0	0	0	0	0	–	–	–
Hajihassani et al. [15] Comp	IRN	57	CBCT M	74.7	4.7	16.3	0	4.7	0	0	0	0	100	0	0
		43	F	80.7	1.8	7.0	0	8.8	1.8	0	0	0	100	0	0
Martins et al. [22] Comp	PRT	331	CBCT M	94.3	0.6	2.1	0.6	1.5	0	0	0	0.9	99.7	0.3	0
		527	F	96.6	0.9	0.8	0.4	1.3	0	0	0	0	100	0	0
Alfawaz et al. [2] Comp	SAU	172	CBCT M	90.1	5.3	0.6	3.5	0.6	0	0	0	0	95.9	4.1	0
		171	F	90.1	3.5	0	1.7	1.2	0	0	3.5	0	95.3	3.5	1.2
Mashyakhy and Gambarini [24] Comp	SAU	188	CBCT M	94.7	0	2.7	0	1.6	0	0	0	1.1	100	0	0
		191	F	99.0	0	0.5	0	0	0	0	0	0.5	100	0	0
Rajakeerthi and Nivedhitha [33] Comp	IND	114	CBCT M	55.3	8.8	6.1	4.4	15.8	3.5	1.8	4.4	0	89.5	7	3.5
		86	F	57.0	7.0	8.1	5.8	9.3	2.3	5.8	4.7	0	90.7	5.8	3.5
Shemesh et al. [47]	ISR	M 831	CBCT	96.4	0.6	1.7	0.1	0.6	0	0	0.1	0.5	99.5	0.5	–
		F 847		97.6	0.1	1.1		0.5	0	0	0.3	0.2	99.8	0.2	–
Total	-	3996	M	82,2	3,4	4,9	3,3	4,1	0,8	0,5	0,6	0,5	97,8	1,7	0,6
			F	88,1	2,5	2,3	1,1	3,6	0,5	0,7	1,1	0,2	98,0	1,6	0,9
			Total	85.1	3.0	3.6	2.3	3.8	0.7	0.6	0.8	0.3	97.9	1.6	0.7

The root canal configurations are given according to the classifications of Weine et al. (We) [50], Vertucci (Ve) [40] and Briseño Marroquín et al. (Br) [44]

*Comp* comparative study design, *Met* research methodology employed, *PP* country 3-digit code of population investigated, * no classification given/possible, *Cl* clearing method, *In* India ink dye, *CBCT* cone-beam computed tomography, *F* female, *M* male

The meta-analysis of seven studies sorted by geographical location by continent demonstrated that a significantly higher number of RCC type 1–2–1/1 (OR [95%CI] = 2.05 [1.27, 3.33]) and 2–2–2/2 (OR [95%CI] = 2.32 [0.65, 8.36]) were observed in male than in female patients (Additional file 1: Fig. S1, Additional file 2: Fig. S2, Additional file 3: Fig. S3, Additional file 4: Fig. S4, Additional file 5: Fig. S5).

## Discussion

Several different research techniques have been used to examine the root canal morphology of different teeth types; however, to the best of our knowledge, the mandibular second premolar (Mn2P) has not been investigated by means of micro-computed tomography (micro-CT), which has been referred to as the gold standard research method [49]. It has been reported [48] that micro-CT has proven to be a reproducible, non-destructive and accurate high-resolution method when investigating the internal morphology of root canals. Although CBCT does not provide a similar high-resolution root canal morphology detail when compared with micro-CT [21], its use for this purpose is relatively widespread. More than half of the reviewed studies in this report (Table 1) were performed by means of CBCT [2, 3, 7, 9–12, 15–24, 26, 27, 29, 33–37, 42, 47]. This type of imaging was introduced by Yu et al. [42] for the investigation of Mn2Ps. Advantages of CBCT are that it allows large sample sizes, it can be performed in vivo, and is relatively fast; in addition, intra-observer variances have not been observed [9, 21]. Other root canal morphology research methods such as clearing [4, 8, 14, 17, 28, 32, 36, 39, 40], cross-Secs. [5, 38] or radiographic examinations [5, 25, 30, 43, 46] have also been employed, yet less frequently.

The Mn2Ps sample sizes in this review varied from 40 [28] to 1678 [47] teeth; however, most of the included studies had a sample size higher than 100 teeth. In studies in which the number of roots of Mn2Ps was reported, the predominant type was single-rooted (89.5–100%) [2, 3, 6, 7, 10, 11, 14–16, 18, 20–24, 27–29, 31–35, 37–39, 41, 42, 46, 47]. Two roots were reported in 0.1–8% [2, 3, 6, 7, 29, 38, 39, 41, 47] of the reviewed studies; these are the lowest and highest ones reported by Martins et al. [20, 21, 23] and Singh and Pawar [39], respectively. Three-rooted Mn2Ps were seldom reported in 0.1–3.5% [2, 7, 33]. Rajakeerthi and Nivedhitha [33] report a relative higher incidence of three-rooted Mn2Ps (3.5%) and RCCs in an Indian population.

Overall, Vertucci’s [40] RCC is by far the most commonly used system in the studies included in this review; all but five of the reviewed studies [13, 30, 34, 38, 43] used this RCC assessment method. In all studies examined (Table 1), Briseño’s Marroquín et al. [44] 1–1–1/1 RCC (Vertucci’s and Weine’s Type I) is the one most frequently reported (55.3–99.6%) with the exception of Bürklein et al. [7], where, by means of CBCT imaging, the 1–1–2/2 RCC (Vertucci’s Type V) was the most frequently observed one out of 871 Mn2Ps (57.1%). Contrary to other findings, these authors reported the lowest 1–1–1/1 (Vertucci’s and Weine’s Type I) RCC frequency (39%) when compared with the other studies reviewed. In approximately half of the reviewed studies, a 1–1–2/2 RCC (Vertucci’s Type V) was the second most frequently observed one in Mn2Ps; yet, a high frequency range between 0.5 and 57.1% was reported [4, 6–8, 15, 17–21, 23, 28, 29, 33, 35, 37, 38, 40, 42]. Salarpour et al. [35] observed, also by means of CBCT, a relative high frequency (22%) of the 1–1–2/2 (Vertucci’s Type V) RCC. Bulut et al. [6] investigated 549 Mn2Ps by means of CBCT in a Turkish population and reported that 98.5% of the sample had a 1–1–1/1 (Vertucci’s and Weine’s Type I) followed by only 0.5% with a 1–1–2/2 (Vertucci’s Type V) RCC.

Approximately one third of the reviewed studies showed a 2–2–2/2 RCC (Vertucci’s Type IV; Weine’s Type III) as the second most common RCC with a frequency ranging between 0.6 and 18% [10, 11, 13, 14, 22, 25, 26, 30, 31, 34, 36, 43, 46]. Within this RCC, Sert and Bayirli [36] reported the highest frequency (18%) in a male group. The 2–2–1/1 RCC (Vertucci’s and Weine’s Type II) has often been reported, mostly with a relative low frequency (0.1–10.8%) [2–6, 9, 14, 15, 17, 18, 21–24, 26, 31–33, 36, 37, 42, 43, 47]; however, Singh and Pawar [39] reported by far the highest 2–2–1/1 RCC frequency (30.0%) in Mn2Ps. Among all the summarized studies (Table 1), other RCCs such as 1–2–1/1, 2–1–2/2, 1–2–1/2 and 1–1–3/3 (Vertucci’s Types III, VI, VII, VIII) were reported less frequently.

Four of the reviewed studies [13, 25, 30, 43] appeared before Vertucci’s [40] classification was published. Pineda and Kuttler [30], Green et al. [13] and Myioshi et al. [25] only reported one and two root canals in Mn2Ps and their results have been tabulated (Table 1] according to Vertucci’s [40], Weine’s et al. [50] and Briseño Marroquín’s et al. [44] RCCs. Within the results of these studies [13, 25, 30], a single root canal was categorized as a Vertucci’s and Weine’s Type I and Briseño Marroquín’s 1–1–1/1 RCC. Two root canals correspond with Vertucci’s Type IV, Weine’s Type III and Briseño Marroquín’s 2–2–2/2 RCC. A single root canal was reported between 92 and 98.8% and two root canals between 1.2 and 8% by these authors [13, 25, 30] in Mn2Ps. By contrast, Zillich and Dowson [43] reported an additional RCC (Vertucci’s and Weine’sType II and Briseño Marroquín’s 2–2–1/1 RCC) where two individual root canals merge and exit together at the apical main foramen. The fact that the publications [13, 25, 30, 43] prior to the one of Vertucci [40] only distinguished between one or two root canals may influence the 2–2–2/2 RCC (Vertucci’s Type IV; Weine‘s Type III) frequency estimation among the reviewed studies; consequently, this RCC in Mn2P should therefore be considered with caution.

A total of twelve studies included in this review [2, 5, 15, 17, 19, 21, 22, 24, 29, 33, 36, 47] are comparative; gender differences were examined in seven studies [2, 15, 22, 24, 33, 36, 47]. All gender comparative studies, with the exception of Sert and Bayirli [36], were carried out by means of CBCT imaging. The gender comparative studies report a higher single root canal frequency in females, with the exception of Alfawaz et al. [2], who report equal frequencies in both genders. The majority of reported Mn2Ps were single-rooted (89.5–100%) in both genders, followed by a contrasting lower frequency of two-rooted Mn2P (0–7%). Only Alfawaz et al. [2] and Rajakeerthi and Nivedhitha [33] reported 1.2 and 3.5% of three-rooted Mn2Ps, respectively. These authors reported that a 1–1–1/1 RCC (Vertucci’s and Weine’s Type I) was the most frequently one observed in both genders; however, this RCC was more frequently observed in women (84.7%) than in men (77.6%) (Table 2). Sert and Bayirli [36] examined, by means of the clearing technique, 200 Mn2Ps and reported a relative high variability between the male and female groups. A 1–1–1/1 RCC (Vertucci’s and Weine’s Type I) was the one most frequently observed in both male (57%) and female (85%) groups, followed by a 2–2–2/2 RCC (Vertucci’s Type IV; Weine’s Type III) in the male (18%) and a 1–1–2/2 RCC (Vertucci’s Type V) in the female (8%) group. The authors report an 18% 2–2–2/2 RCC (Vertucci’s Type IV; Weine’s Type III) frequency in the male group while this RCC was not observed in any of the female individuals.

Different root canal morphology research methods have also been compared. Khademi et al. [17] compared results from 182 mandibular first and second premolars with the clearing and CBCT techniques and reported that 87% of the results were in agreement with both research techniques. The highest agreement rate observed was in the 2–2–2/2 RCC (Vertucci’s Type IV; Weine’s Type III) and the lowest one in the 1–1–2/2 RCCs (Vertucci’s Type V) groups. According to the authors, the CBCT technique demonstrated a higher accuracy than the clearing technique when recognizing C-shaped root canals but a lower accuracy in the recognition of lateral canals. Bolhari et al. [5] reported an agreement of 96.77 to 98.62% between bucco-lingual as well as mesio-distal projected radiographs and the cross-section technique. Regarding different ethnic groups, the comparative study Pedemonte et al. [29] reported that the 1–1–1/1 RCC (Vertucci’s and Weine’s Type I) was the most frequent one observed in Mn2Ps in Belgian (92.1%) and Chilean (95.0%) populations. Martins et al. [21] compared by means of CBCT the data obtained from a Chinese and a west European population and reported a slightly higher 1–1–1/1 RCC (Vertucci’s and Weine’s Type I) frequency in the Mn2Ps of the Chinese (99.6%) than in the west European (95.7%) groups. Using radiography, Trope et al. [57] investigated the RCC frequencies in 400 Mn2Ps in different ethnic groups and reported, at that time, that an Afro-American ethnic group (7.8%) had more than one canal more frequently than a Caucasian ethnic group (2.8%); however, these differences were not statistically significant. Yet, this study did not meet the inclusion criteria since it does not distinguish between different RCCs, and it was not included in the current systematic review. A comparative study [19] regarding different individual ages reported a slight 1–1–1/1 RCC (Vertucci’s and Weine’s Type I) decline from younger to older age groups (98.8% [21–40 years], 96.2% [41–60 years] and 92.5% [≥ 61 years]).

Although most Mn2Ps are single-rooted teeth, caution should always be exercised when attempting to compare the internal root canal morphology between different investigations since some authors do not report the number of roots observed. This precaution can be illustrated with Briseño Marroquín’s et al. [44] 2–2–2/2 RCC, which describes the root canal morphology of one particular root, whereas Vertucci’s [40] and Weine’s et al. [50] classifications consider the tooth with its roots as a single entity.

## Conclusions

- Mandibular second premolars are most frequently single-rooted (89.5–100%).
- The 1–1–1/1 RCC (Vertucci’s and Weine’s et al. Type I) is the most frequently observed one, followed by a 1–1–2–/2 (Vertucci’s Type V) and a 2–2–2/2 RCC (Vertucci’s Type IV; Weine’s Type III).
- Meta-analysis of studies investigating gender differences report a significantly higher number of RCC type 1–2–1/1 and 2–2–2/2 in male than in female individuals.
- CBCT imaging is nowadays the research method most frequently employed in Mn2Ps morphological investigations.
- Although most Mn2Ps are single rooted with a single canal (1–1–1/1), the possibility of more complicated RCCs should always be considered when planning and performing an endodontic treatment.

## Supplementary Information

**Additional file 1**: **Fig. S1**. Quantitative meta-analyses for RCC type 1–1–1/1. Odds Ratio (OR) (and 95% confidence intervals (95%CI)) was used to calculate differences between patient’s sex. Forest plots, heterogeneity parameter (I^2^) as well as overall statistics (Z, P) are given

**Additional file 2**: **Fig. S2**. Quantitative meta-analyses for RCC type 1–1–2/2. Odds Ratio (OR) (and 95% confidence intervals (95%CI)) was used to calculate differences between patient’s sex. Forest plots, heterogeneity parameter (I^2^) as well as overall statistics (Z, P) are given

**Additional file 3**: **Fig. S3**. Quantitative meta-analyses for RCC type 1–2–1/1. Odds Ratio (OR) (and 95% confidence intervals (95%CI)) was used to calculate differences between patient’s sex. Forest plots, heterogeneity parameter (I^2^) as well as overall statistics (Z, P) are given

**Additional file 4**: **Fig. S4**. Quantitative meta-analyses for RCC type 2–2–1/1. Odds Ratio (OR) (and 95% confidence intervals (95%CI)) was used to calculate differences between patient’s sex. Forest plots, heterogeneity parameter (I^2^) as well as overall statistics (Z, P) are given

**Additional file 5**: **Fig. S5**. Quantitative meta-analyses for RCC type 2–2–2/2. Odds Ratio (OR) (and 95% confidence intervals (95%CI)) was used to calculate differences between patient’s sex. Forest plots, heterogeneity parameter (I^2^) as well as overall statistics (Z, P) are given

## Data Availability

All data generated or analyzed during this study are included in this published article and its supplementary files.
