# Supplementary figures and images for: Root canal morphology of the mandibular second premolar: a systematic review and meta-analysis
Source: BMC Oral Health. 2021 Jun 16;21:309. doi: 10.1186/s12903-021-01668-z (PMC8207603; doi:10.1186/s12903-021-01668-z)

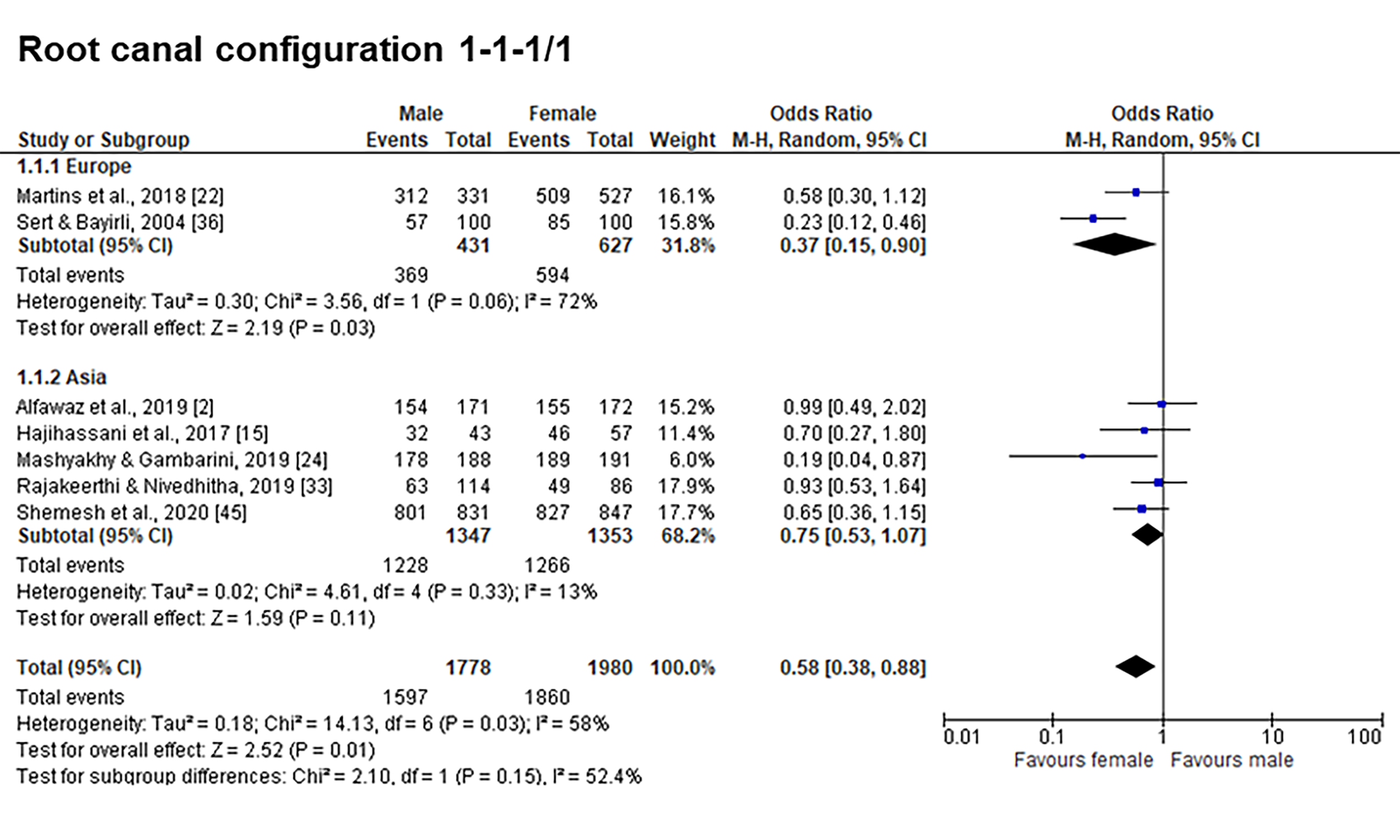

Supplement: Supplementary file 1 — Additional file 1: Fig. S1. Quantitative meta-analyses for RCC type 1–1–1/1. Odds Ratio (OR) (and 95% confidence intervals (95%CI)) was used to calculate differences between patient’s sex. Forest plots, heterogeneity parameter (I2) as well as overall statistics (Z, P) are given [file 12903_2021_1668_MOESM1_ESM.tif]

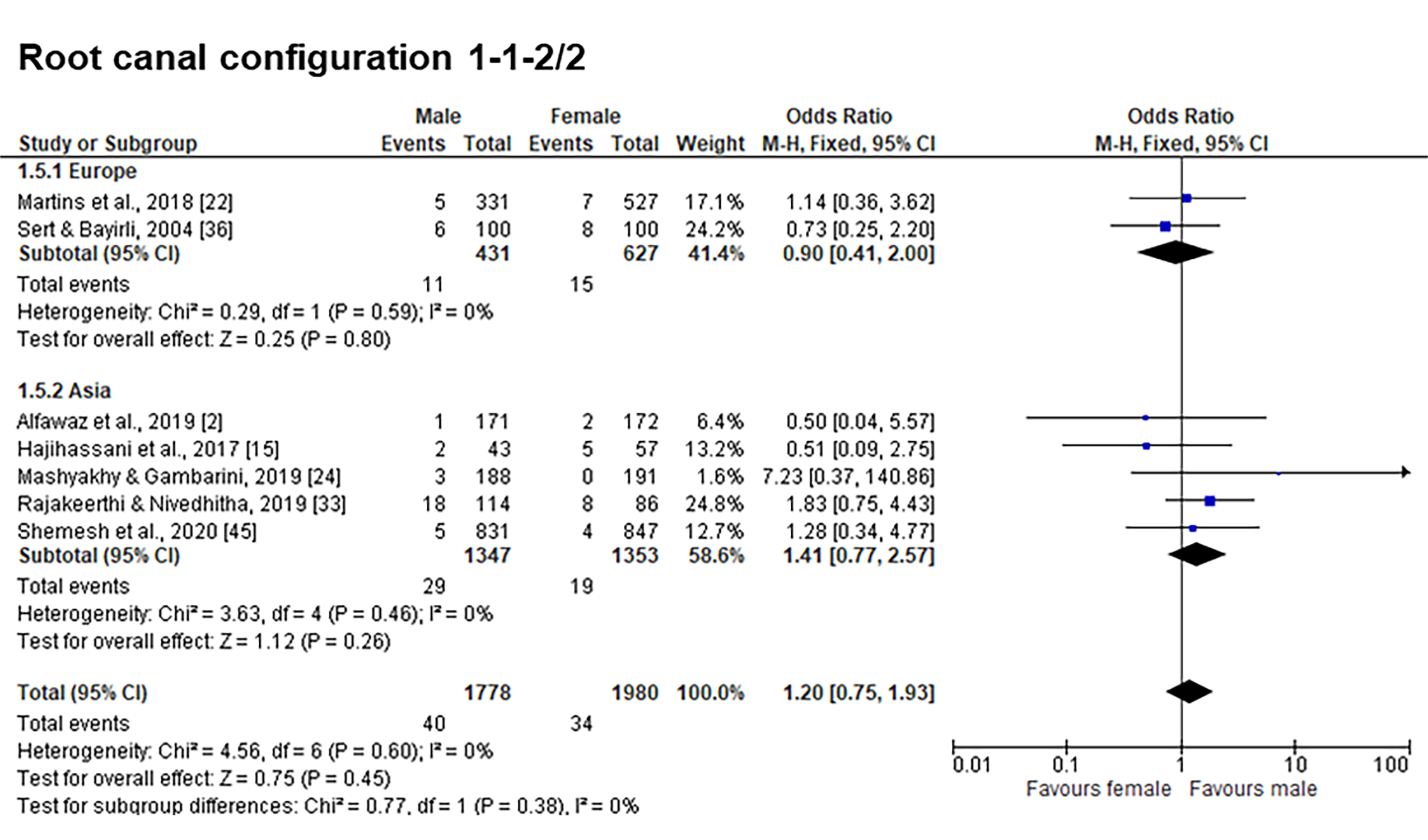

Supplement: Supplementary file 2 — Additional file 2: Fig. S2. Quantitative meta-analyses for RCC type 1–1–2/2. Odds Ratio (OR) (and 95% confidence intervals (95%CI)) was used to calculate differences between patient’s sex. Forest plots, heterogeneity parameter (I2) as well as overall statistics (Z, P) are given [file 12903_2021_1668_MOESM2_ESM.tif]

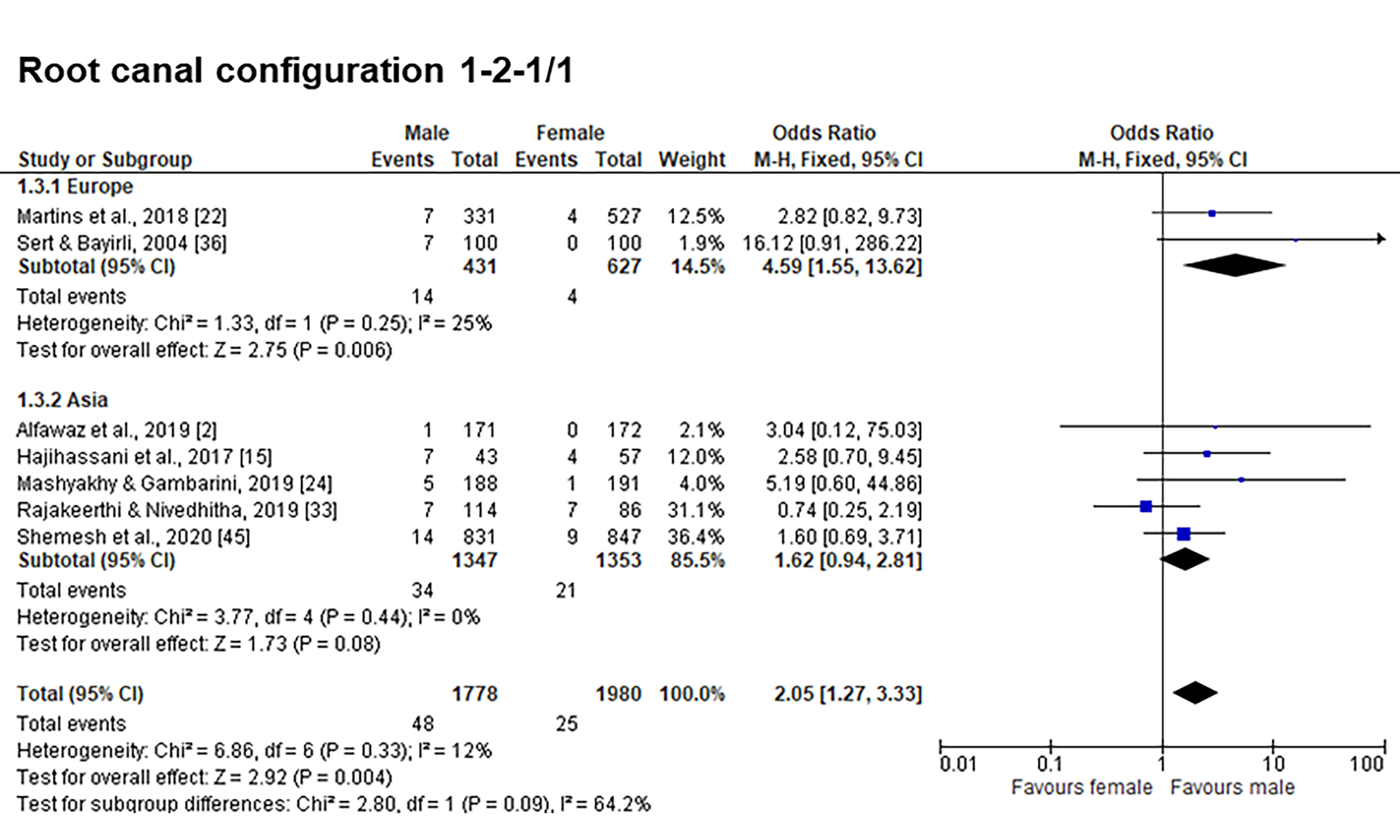

Supplement: Supplementary file 3 — Additional file 3: Fig. S3. Quantitative meta-analyses for RCC type 1–2–1/1. Odds Ratio (OR) (and 95% confidence intervals (95%CI)) was used to calculate differences between patient’s sex. Forest plots, heterogeneity parameter (I2) as well as overall statistics (Z, P) are given [file 12903_2021_1668_MOESM3_ESM.tif]

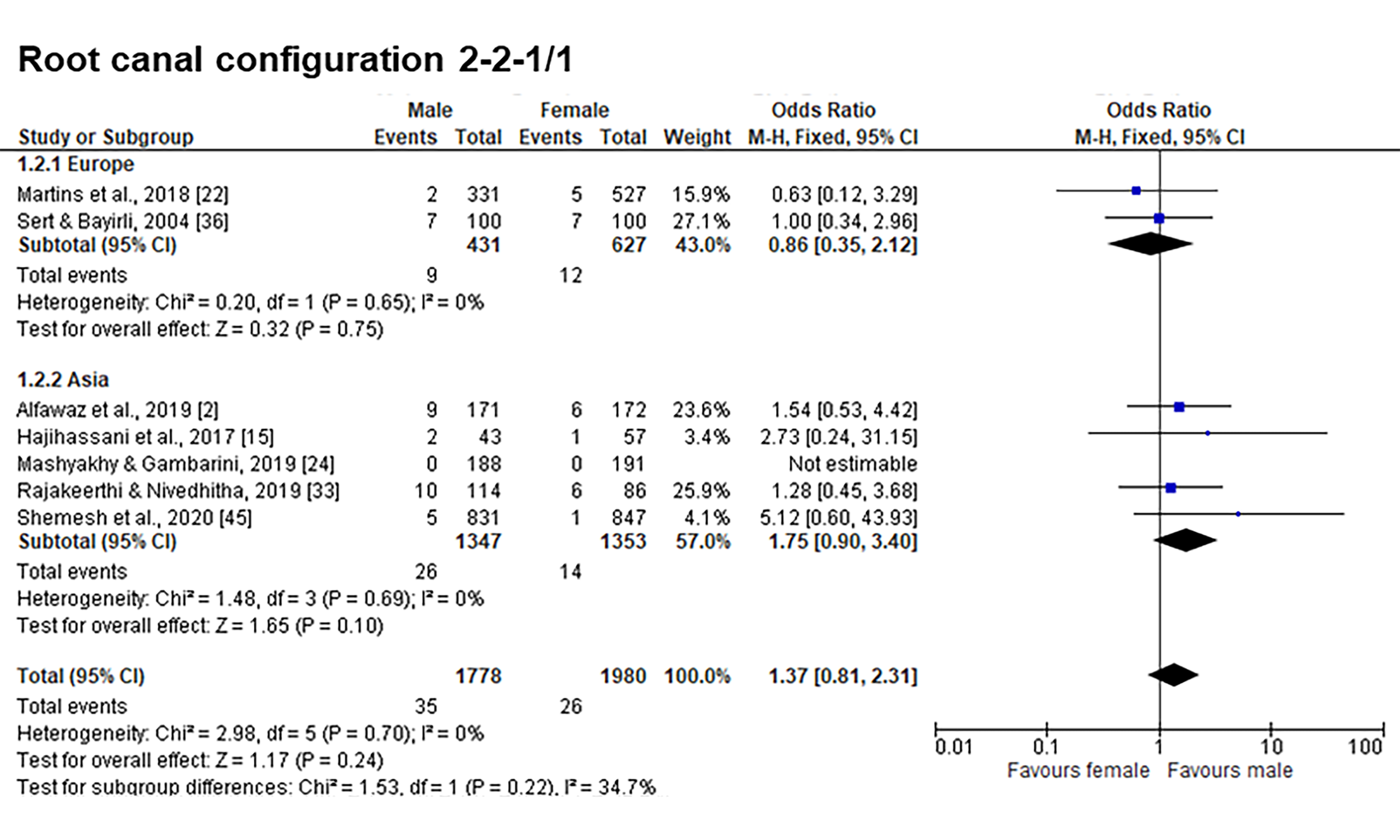

Supplement: Supplementary file 4 — Additional file 4: Fig. S4. Quantitative meta-analyses for RCC type 2–2–1/1. Odds Ratio (OR) (and 95% confidence intervals (95%CI)) was used to calculate differences between patient’s sex. Forest plots, heterogeneity parameter (I2) as well as overall statistics (Z, P) are given [file 12903_2021_1668_MOESM4_ESM.tif]

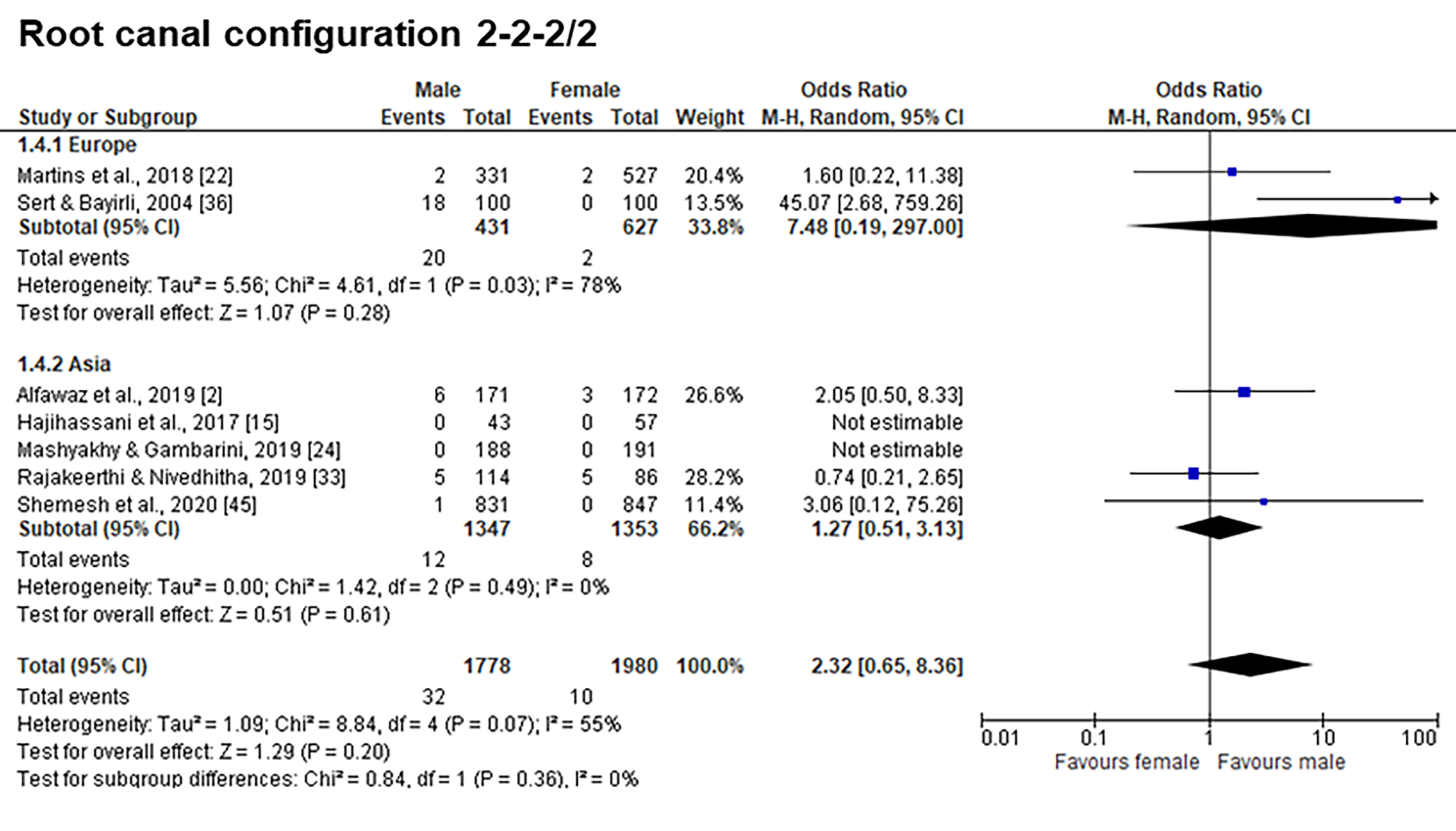

Supplement: Supplementary file 5 — Additional file 5: Fig. S5. Quantitative meta-analyses for RCC type 2–2–2/2. Odds Ratio (OR) (and 95% confidence intervals (95%CI)) was used to calculate differences between patient’s sex. Forest plots, heterogeneity parameter (I2) as well as overall statistics (Z, P) are given [file 12903_2021_1668_MOESM5_ESM.tif]
